# Efficacy of a mandibular advancement intraoral appliance (MOA) for the treatment of obstructive sleep apnea syndrome (OSAS) in pediatric patients: A pilot-study

**DOI:** 10.4317/medoral.22580

**Published:** 2018-11-21

**Authors:** Gabriela Modesti-Vedolin, Caroline Chies, Simone Chaves-Fagondes, Eduardo Piza-Pelizzer, Márcio Lima-Grossi

**Affiliations:** 1 DDS, MS, PhD, Post-Graduate Program in Dentistry (Prosthodontics), Faculty of Dentistry, Pontifical Catholic University Universidade Católica do Rio Grande do Sul (PUCRS). Av. Ipiranga, 6681, 90619-900, Porto Alegre, RS, Brazil; 2 DDS, Faculty of Dentistry, Pontifical Catholic University Universidade Católica do Rio Grande do Sul (PUCRS). Av. Ipiranga, 6681, 90619-900, Porto Alegre, RS, Brazil; 3 MD, PhD, Specialist in Sleep Medicine, Pneumology Center, Sleep Laboratory, Clinical Hospital of Porto Alegre (HCPA), Rua Ramiro Barcelos, 2350, sala 2050, 90035-903, Porto Alegre, RS, Brazil; 4 DDS, MS, PhD, Professor, Post-Graduate Program in Dentistry (Prosthodontics and Implantology), Araçatuba Faculty of Dentistry, University of the State of São Paulo (UNESP), Brazil; 5 DDS, MS, PhD, Professor, Post-Graduate Program in Dentistry (Prosthodontics), Faculty of Dentistry, Pontifical Catholic University of Rio Grande do Sul (PUCRS). Av. Ipiranga, 6681, 90619-900, Porto Alegre, RS, Brazil

## Abstract

**Background:**

To evaluate the treatment efficacy of a mandibular advancement intraoral appliance (MOA) for treatment of obstructive sleep apnea syndrome (OSAS) in pediatric patients.

**Material and Methods:**

Eighteen patients (mean=8.39 years old, women=44.4%) were selected. Sleep disorders, sleep bruxism, and temporomandibular disorders were assessed by the Sleep Disturbance Scale for Children (SDSC), the BiteStrip® (portable SB device), and the Research Diagnostic Criteria for Temporomandibular Disorders, respectively. The clinical diagnosis of OSAS was confirmed with a type 3 portable monitor device (ApneaLinkTM Plus). A silicon-based material MOA was used by patients for 60 days, and the results were compared to baseline.

**Results:**

The median RDI was significantly reduced from 10 to 4.5 events/hour. Nadir SpO2 significantly increased from 82.6% to 88.9%. Total snoring events/hour have also significantly decreased from 205.5 to 91.5. Signs and symptoms of TMD remained unaltered. There was also a reduction from moderate to absence of SB in 12 patients. Similarly, all variables measured by the SDSC have had very significant reductions: disorders of initiating and maintaining sleep, sleep disordered breathing, disorders of arousal, nightmares, sleep wake transition disorders, disorders of excessive somnolence, and sleep hyperhidrosis.

**Conclusions:**

In selected cases, OA maybe considered as an alternative for the OSAS treatment.

** Key words:**Snoring appliances, sleep apnea, bruxism, sleep disorders, children

## Introduction

Obstructive sleep apnea syndrome (OSAS) is a respiratory disorder, common in children, which occurs during sleep; characterized by prolonged partial obstruction of the upper airway space (hypoventilation) and/or by intermittent complete obstruction (apnea). This process interrupts both the normal ventilation, and the upper airway flow, and the normal sleep patterns (1). The main symptoms of OSAS are: snoring, respiratory effort, and intense body movements during sleep. These variables have serious consequences to children: slowing down in normal body growth, and eventual cardiovascular complications such as pulmonary hypertension. The major risk factors for OSAS are: obesity, craniofacial malformations, and neuromuscular diseases. The prevalence of childhood snoring, based on clinical history/examination and structured questionnaires, varies from 1.5 to 15% (2). The OSAS pathophysiology is multifactorial, with many anatomical/functional/neuromuscular factors involved. Soft/adipose tissues, musculature, and the craniofacial bones will directly affect the configuration and dimension of the pharynx. Consequently, it is frequently observed that patients with OSAS present with: hypotonic tongue, macroglossy, retrognathic mandible/maxilla, micrognathism, V-shaped palate, narrow arches, and crossbites (3,4).

Due to its chronic nature, OSAS treatments consist of clinical and surgical modalities, depending on its severity. The treatment objectives are both to normalize breathing during sleep, and to eradicate daytime sleepiness, and to reduce neuropsychological/cardiovascular alterations. It must provide the patient with good quality of life without risks or side effects (5,6). The mandibular advancement intraoral appliance (MOA) therapy is used during sleep with the objective of preventing the collapse between the oropharynx and the tongue base (i.e., the upper airway space patency). They are non-invasive, comfortable, easily adaptable, and effective devices for patients; and they have been a growing line of treatment for the last 20 years (6). The MOA primary mode of action is to advance the mandible and to reposition the tongue, with the objective of increasing the airway space and to facilitate the superior respiratory system (7). Pediatric studies using MOA are missing, and there are few studies using this appliance during sleep for OSAS in this age group (8).

The primary objective of this study was to evaluate the effects of the MOA treatment in children with OSAS. In addition, the effects of MOA in sleep bruxism (SB) and signs and symptoms of temporomandibular disorders (TMD) will also be assessed.

## Material and Methods

-Study design

A before-and-after clinical trial design was carried out with the objective to assess the improvement in upper airway obstruction after the use of MOA in children with OSAS (9).

-Population, inclusion and exclusion criteria, and study protocol

Eighteen patients from the Otolaryngology Services at the São Lucas Hospital and the Clinical Hospital of Porto Alegre, who were in the waiting list for amygdalectomy, participated in the study. Patients with clinical history of snoring during sleep (minimum 3 episodes/week), from both sexes, and between the ages of 5 to 12 were included.

Clinical history of sleep apnea and snoring reported by parents, TMD, joint pain, muscle pain, sleep disorders, SB, and daily habits were assessed. Regarding general health, history of systemic diseases and use of medication were also verified. In the clinical examination; the presence of wearing facets, edentations in the lips, tongue and jugal mucosa, and teeth number were also evaluated.

The exclusion criteria were patients with: a) relevant craniofacial skeletal abnormalities, b) history of orthodontic treatment, c) active periodontal disease and/or tooth mobility, d) medication use acting in the central nervous system (anxiolytics/antidepressants), e) unstable occlusion (i.e., without maximum intercuspal position), and f) presence of TMD spontaneous pain.

**Figure 1:**
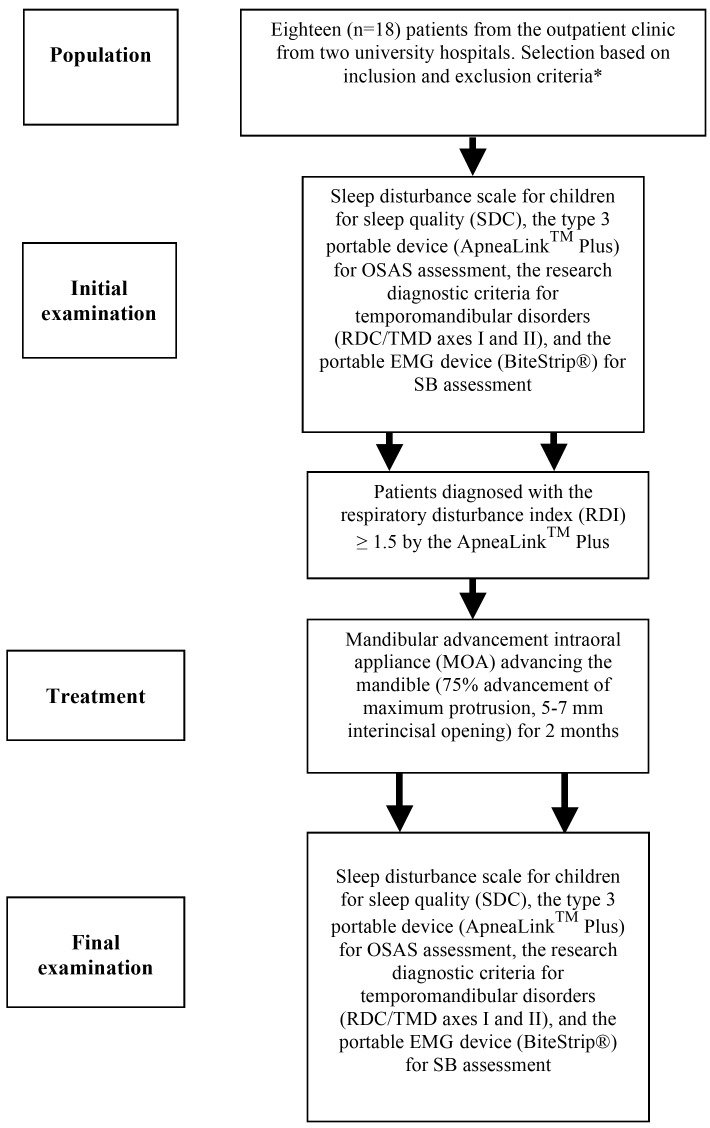
Study diagram describing the research protocol. * Inclusion criteria: patients who were in the waiting list for amygdalectomy, with clinical history of snoring during sleep (minimum 3 episodes/week), from both sexes, between the ages of 5 to 12.

The following diagnostic tests and questionnaires were used in the OSAS, quality of sleep, SB, and signs and symptoms of TMD assessments: a) respiratory disturbance index (RDI) and blood oxygen saturation (SpO2) using a type 3 portable device (ApneaLinkTM Plus, version 9.00, ResMed), d) the Sleep Disturbance Scale for Children (SDSC) self-reported by parents, e) the portable electromyogram (EMG) device for SB (BiteStrip®), and f) the Research Diagnostic Criteria for Temporomandibular Disorders (RDC/TMD) axes I and II (Fig. 1). Only in included patients presenting RDI≥1.5 in the ApneaLinkTM Plus, all above tests were performed before-and-after the 60-day use of the MOA for 2 months.

-Obstructive sleep apnea evaluation by a portable monitoring device and by subjective sleep assessment

In the preliminary diagnosis of OSAS, parents answered both the SDSC, and the parents’ reports regarding snoring or any sleep alterations (10). The SDSC evaluates children’s sleep patterns of behavior. The SDSC is reproducible, valid, and with internal consistency; and it has the capacity to distinguish common sleep disorders among children and adolescents: a) disorders of initiating and maintaining sleep, b) sleep breathing disorders, c) disorders of arousal/nightmares, d) sleep-wake transition disorders, e) disorders of excessive somnolence, and f) sleep hyperhidrosis (11).

In order to confirm the OSAS diagnosis, these patients used a portable home cardiorespiratory monitoring type 3 device according to the AASM (12,13). This device (ApneaLinkTM Plus, version 9.00, ResMed) has 4 monitored channels, including respiratory effort, pulse rate, and peripheral oxygen saturation (SpO2) and has been validated against polysomnography (14-18). The variables measured by the ApneaLinkTM Plus, as recommended by the AASM, were: a) oxygen desaturation index (ODI) calculated from the number of SpO2 drops below 3% from baseline saturation, b) RDI calculated from the number of obstructive episodes over the total recording time, c) snoring events, d) saturation time, e) average saturation, f) lowest saturation, and g) registration time (14).

Patients underwent an initial portable apnea/hypopnea appliance evaluation which confirmed the initial clinical diagnosis. Then, only patients presenting RDI≥1.5 in the ApneaLink™ Plus used the individualized MOA for 2 months with the objective of reducing obstructive events, and underwent another global evaluation (before-and-after) (5,19). All procedures were carried out by a single experient and trained examiner; and the exam interpretation was always performed by the same physician, who was a sleep medicine specialist blind to the use of the MOA, following the AASM scoring guidelines (2012) (14). The cardiovascular registrations were performed during the whole night, in non-induced sleep, at the patients’ home place.

-Mandibular advancement intraoral appliance manufacturing

In order to make the MOA appliance, type IV gipsy casts were mounted in a semi-adjustable articulator (Bio-Art Dental Equipments Ltd., São Paulo, Brazil) at 70% of the patient’s mandibular maximum protrusive movement (8 mm advancement), with a mandibular opening varying from 5 to 7 mm. Then, two soft, 3 mm thick, translucent thermoplastic bite splints were made in the thermo-vacuum device (Plastvac P7, Bio-art Dental Equipments, São Paulo, Brazil). The splints were fused in the articulator in the preregistered position using a micro torch (Piezo Electronic Micro Torch-GB 2001, Micro Torch-Blazer) (18).

-Sleep bruxism assessment

Along with the valid portable respiratory evaluation, patients used a validated against polysomnography disposable and portable EMG device (BiteStrip®) during sleep for SB assessment (20). The BiteStrip evaluates the number of SB episodes by the registration of the left masseter EMG during 5 hours of sleep time. After utilization, an electrochemical display shows values between 0 to 3 (e.g., 0 = no bruxism, ≤ 39 episodes; 1 = mild bruxism, 40 - 74 episodes; 2 = moderate bruxism, 75 - 124 episodes; 3 = severe bruxism, ≥ 125 episodes; and E = error message) (21).

-Criteria for temporomandibular disorders assessment

Selected patients underwent the clinical examination for assessment of signs and symptoms of TMD with the validated Brazilian Portuguese version of the RDC/TMD axes I and II (22). The clinical examination was performed by the same examiner, who did not participate in the selection and portable instruments application, following the guidelines of the RDC/TMD axis I (http://www.rdc-tmdinternational.org/TMDAssessmentDiagnosis/RDC-TMD/Translations/Portuguese(Brazil).aspx). The variables analyzed were: a) disability points (DP), b) chronic pain grade (CPG), c) characteristic pain intensity (CPI), d) muscle disorders (group I), e) disk displacement (group II), and f) temporomandibular joint arthralgia/osteoarthrosis/osteoarthritis (group III).

-Statistical analysis and sample size calculation

For the statistical analysis, the SPSS v. 20.0 (SPSS, Chicago, Illinois, USA) was used. The Shapiro-Wilk test was used for normality testing. Wilcoxon and Paired Student’s t tests were used for before-and-after evaluation (*p* < 0.05). The sample size calculation, comparing two proportions (confidence level = 95%, type I error = 0.05, type II error = 0.2, expected difference between p1 and p2 = 40%) yielded a sample = 20 (23).

## Results

-Social and demographic description of the population

Out of 20 subjects who carried out the first portable study, 2 did not perform the second evaluation (i.e., 10% drop out rate). One patient did not tolerate the maxillary/mandibular arch alginate impression, and the other felt discomfort during the first device. The final sample (n = 18) was comprised predominantly by children (mean age = 8.3±2.3 years, range = 5-12 years), from both sexes (55.6% males, 44.4% females), in the elementary school (66.7%) and pre-school (33.3%) levels, and predominantly from low income families with income up to 3 minimum wages per month (94.4%).

-Obstructive sleep apnea syndrome evaluation by a portable monitoring device

In  Table 1, the cardiovascular portable monitoring examination with the ApneaLinkTM Plus has shown an improvement in most variables after the MOA use. The median ODI has shown a sharp and significant reduction (33.3%, *p* < 0.01). The mean RDI reduced significantly in all patients of our sample (55%, *p* < 0.001). The number of snoring events has also declined with the treatment (55.5%, *p* < 0.001). The average oxygen saturation also had a significant reduction (*p* < 0.05). The Nadir SpO2 significantly increased in 7.6% after the MOA use (*p* < 0.05). The time with oxygen saturation below 90% (% SpO2 < 90%) and the registration time have also declined, but they were non-significant.

**Table 1:**
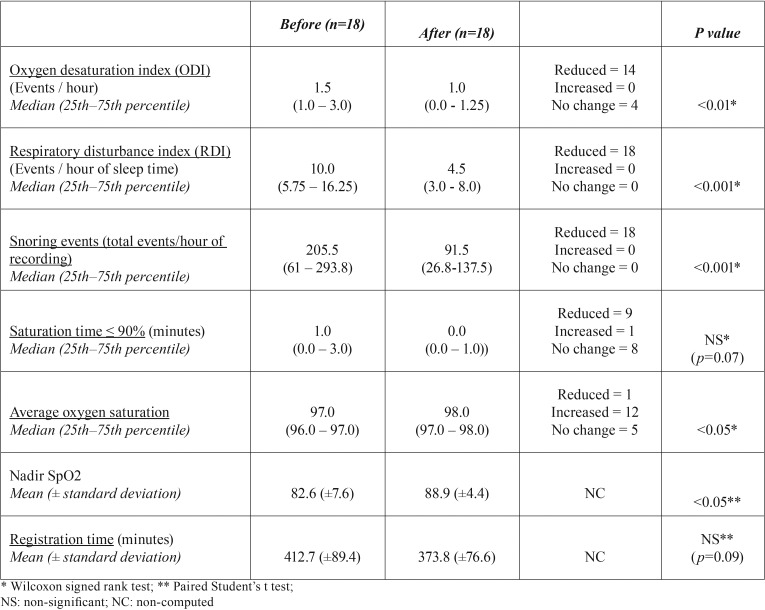
Portable monitoring (ApneaLinkTM Plus, version 9.00, ResMed) before and after the use of a mandibular advancement intraoral appliance (MOA) in children between 5 to 12 years diagnosed with sleep apnea obstructive syndrome (OSAS).

-Signs and symptoms of temporomandibular disorders and sleep bruxism

In  Table 2, the signs and symptoms of TMD did not increase after the use of the MOA. On the contrary, some RDC/TMD axis II variables have shown significant reduction: chronic pain grade (*p* <0.01) and characteristic pain intensity (*p* < 0.05). Disability points have shown no change, and it was non-significant. In the RDC/TMD axis I variables, only muscle disorders were present, and they have also shown a tendency towards reduction (20%), but they were non-significant. Neither disk displacements nor TMJ arthralgia/osteoarthritis/osteoarthrosis disorders were diagnosed in this sample. Regarding the BiteStrip®, a significant and sharp reduction (66%, *p* < 0.01) was observed in SB prevalence.

**Table 2:**
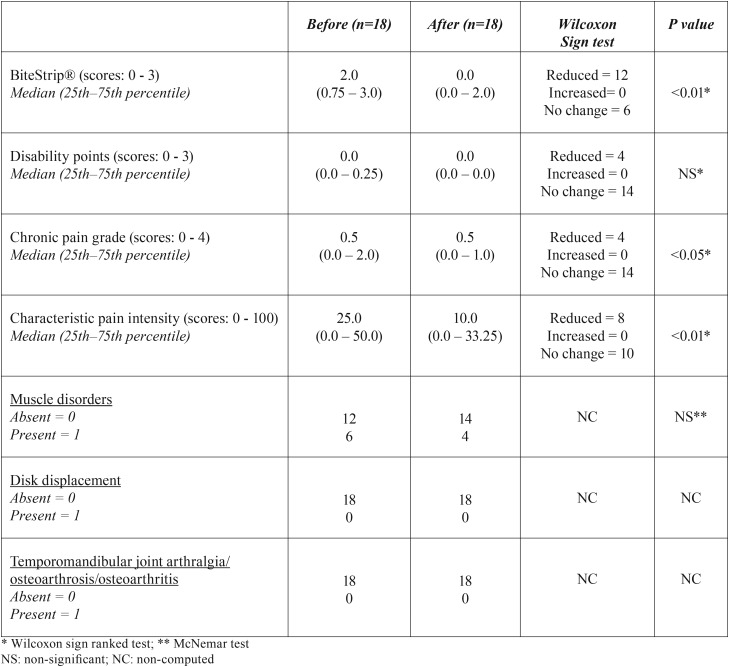
Sleep bruxism assessment (BiteStrip®) and the Research Diagnostic Criteria for Temporomandibular Disorders (RDC/TMD) axes I and II assessment before and after the use of a mandibular advancement intraoral appliance (MOA) in children between 5 to 12 years diagnosed with sleep apnea obstructive syndrome (OSAS).

-The subjective sleep quality improvement

The parents’ subjective report on the SDSC demonstrated a very significant percent reduction/improvement in all analyzed variables: a) disorders of initiating and maintaining sleep (34.5%, *p* < 0.001), b) sleep breathing disorders (37.5%, *p* < 0.001), c) disorders of arousal/nightmares (16.3%, *p* < 0.01), d) sleep wake transition disorders (32.2%, *p* < 0.001), e) disorders of excessive somnolence (19.9%, *p* <0.01), and f) sleep hyperhidrosis (21.8%, *p* < 0.01) ( Table 3).

**Table 3:**
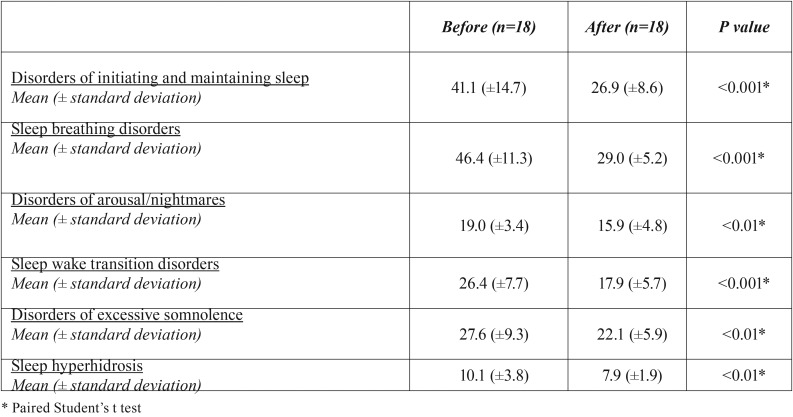
Sleep Disturbance Scale for Children (SDSC) assessment before and after the use of an intraoral appliance in children between 5 to 12 years diagnosed with obstructive sleep apnea syndrome (OSAS).

## Discussion

The MOA has shown here reduced snoring and improved airflow in the treatment of children with OSAS in both subjective (i.e., parents’ self-reported SDSC) and objective (ApneaLinkTM Plus) assessments. This success has already been demonstrated in adults by promoting: the reduction in the daytime sleepiness and in the obstructive episodes during sleep, the improvement in the oxygen saturation, the reduction in snoring intensity and frequency, and the improvement in sleep quality (24). In patients with skeletal/occlusal alterations undergoing orthodontic treatment, improvement in AHI and facial profile has also been demonstrated (25-29). The MOA made here from translucent thermoplastic soft bite splints were flexible, unexpensive, comfortable, easily adaptable, and used by all subjects; they had no interference with dental/skeletal growth within the time period (i.e., 60 days) assessed. However, long-term longitudinal studies should be performed, due to possible irreversible changes in the dentition and TMJs (29). The durability of these soft appliances is also limited, so it is a temporary solution (8,19,20).

Similar to the literature, ApneaLinkTM Plus was an excellent alternative for childrens’ OSAS diagnoses. Only one patient did not tolerate the device, indicating a high compliance by children and treatment acceptance. It can be indicated for monitoring the treatment response with MOA, upper airway surgery, and weight loss (30). The OSAS diagnostic improvement after treatment with the ApneaLinkTM Plus agreed with the results of the SDSC (Brazilian Portuguese version) answered by parents (10,11). The SDSC has also shown improvement in respiratory difficulty during sleep, snoring and sleep apnea; agreeing with the ApneaLinkTM Plus results. However, this cardiovascular monitoring device, different than the overnight in lab PSG, neither assesses sleep macrostructure, nor CO2 measurement (14,15). On the other hand, the device has the capacity to assess air flow, thoracic band, and SpO2 in the patient’s home sleep, which is an advantage in pediatric populations (15).

The subjective SDSC results have also shown significant reduction in grinding sounds after MOA use, agreeing with the objective reduction assessed by the BiteStrip® of SB events. Similar to the literature, MOA has shown significantly greater SB reduction when compared to the Michigan-type bite splint (3,7,8,19,20). On the other hand, MOA cannot replace the Michigan-type bite splint in patients without OSAS, only in those cases where both conditions are in place due to the appliance’s irreversible side effects (20). However, portable EMG cannot replace PSG, and new studies confirming our findings using PSG must be conducted (19,20). This BiteStrip® is indicated for children due to its screening validity, its non-invasive design , and its comfort. In the study sample, muscular and articular disorders (i.e., disk displacements and TMJ pain) were not diagnosed and were not aggravated after the MOA treatment in the short term. In fact, chronic pain grade and characteristic pain intensity had significant reduction.

## Conclusions

Treatment with a soft mandibular advancement intraoral appliance has demonstrated in this study to be effective in the reduction of obstructive sleep apnea and sleep bruxism in a pediatric population in most objective and subjective sleep and sleep bruxism assessments performed. No worsening in signs and symptoms of temporomandibular disorders has been noted.
